# A Turbo Q-Learning (TQL) for Energy Efficiency Optimization in Heterogeneous Networks

**DOI:** 10.3390/e22090957

**Published:** 2020-08-30

**Authors:** Xiumin Wang, Lei Li, Jun Li, Zhengquan Li

**Affiliations:** 1 College of Information Engineering, China Jiliang University, Hangzhou 310018, China; 05a0303091@cjlu.edu.cn (X.W.); P1803085214@cjlu.edu.cn (L.L.); 2 Binjiang College, Nanjing University of Information Science & Technology, Wuxi 214105, China; 3 College of Internet of Things, Jiangnan University, Wuxi 214000, China; lzq722@jiangnan.edu.cn

**Keywords:** energy efficiency, HetNets, eICIC, Q-Learning, reinforcement learning, multistage decision process

## Abstract

In order to maximize energy efficiency in heterogeneous networks (HetNets), a turbo Q-Learning (TQL) combined with multistage decision process and tabular Q-Learning is proposed to optimize the resource configuration. For the large dimensions of action space, the problem of energy efficiency optimization is designed as a multistage decision process in this paper, according to the resource allocation of optimization objectives, the initial problem is divided into several subproblems which are solved by tabular Q-Learning, and the traditional exponential increasing size of action space is decomposed into linear increase. By iterating the solutions of subproblems, the initial problem is solved. The simple stability analysis of the algorithm is given in this paper. As to the large dimension of state space, we use a deep neural network (DNN) to classify states where the optimization policy of novel Q-Learning is set to label samples. Thus far, the dimensions of action and state space have been solved. The simulation results show that our approach is convergent, improves the convergence speed by 60% while maintaining almost the same energy efficiency and having the characteristics of system adjustment.

## 1. Introduction

With the dramatic growing number of wireless devices, more stringent requirements are put forward for performance and energy efficiency of heterogeneous networks (HetNets) [1]. With the increasing complexity of HetNets, the optimization of energy efficiency has more and more challenges and is one of the hot spots of communication network research, especially for the HetNets with 5G BSs. Therefore, in this paper, the efficiency of resource allocation algorithm is studied, in which the reinforcement learning (RL) is utilized and the parameters such as ABS (almost blank sub-frame), CRE (Cell range expansion), and SI-SBSs (sleeping indicaton of small BSs) are jointly considered simultaneously to optimize the energy efficiency of the whole network.

In general, the optimization problems with multi-variables are non-convex NP problems, it is hard to be solved directly. Some can be processed by dividing the original problem into sub-problems which can be iteratively solved with an acceptable complexity. Baidas et al. [2] jointly considered subcarriers assignment and global energy-efficient (GEE) power allocation, and the original problem was divided into two subproblems as subcarrier allocation by many to many matching and GEE maximizing power allocation. By designing a two-stage solution program, the original problem was effectively solved with the ensured stability. Chen et al. [3] jointly investigated the task allocation and CPU-cycle frequency, in order to achieve the minimum energy consumption which scaled down to the sum of two deterministic optimization subproblems by Lyapunov optimization theory. The optimal solutions of the two sub-problems separately which were local computation allocation (LCA) and offloaded computation allocation (OCA) were found to obtain the optimal solution of the upper bound of the original problem. Although decomposition and iteration are efficient to solve the non-convex NP problems in many cases, the complexity of modeling and computing is still high in most cases.

As the AI technologies are developing with a very high speed in the recent years, some learning methods are introduced to solve some complicated optimization problems. As shown in [4,5,6,7,8,9], model-free RL methods can be an efficient way to solve the energy efficiency optimization problem of HetNets, since the precise model process was not necessary. In [4,5], the Actor–Critic (AC) algorithm was applied to optimize energy efficiency of HetNets while the authors did not conduct in-depth research on the selections of basis functions which are challenging for the application of RL. Roohollah et al. [6] introduced a Q-Learning (QL) based distributed power allocation algorithm (Q-DPA) as a self-organizing mechanism to solve the power optimization problem in the networks. In [7], a method based on QL was proposed to solve the energy efficiency and delay problems of smart grid data transmission in HetNets, in which, however, the dimension of action and state space was too large. Ayala-Romero et al. [8,9] combined dynamic programming, neural networks, and data-driven methods to solve problem of energy saving and interference coordination in HetNets.

In this paper, inspired from the previous works [2,4,5,6,7,8,9,10], referring to RL and the idea of converting non-convex NP hard problem into several sub-problems, a turbo QL (TQL) scheme is proposed to optimize energy efficiency in which the traditional QL algorithm is decomposed into several sub-Q-Learning algorithms and has a loop iteration structure, each sub-Q-learning solving each sub-problem. In our scheme, the parameters ABS, CRE, and SI-SBSs are jointly taken into account as action vectors, the user positions are taken as the states in order to fully consider the randomness of users in BSs, and the reward function is designed as a negative reciprocal of the system energy efficiency. The problem of dimensional explosion with increased action space is solved by our proposed TQL structure, and it is acceptable for the complexity of the algorithm. For the states, a fully connected deep neural network is designed to identify state type. The contributions of this paper are summarized as follows.

(1) The reward function is designed as a negative reciprocal of the system energy efficiency to avoid the slow speed of convergence and the possibility of fulling into local optimum. If the magnitude of the reward in RL is too large, it is easy to fall into the local optimization, and too small value can cause the problem of system oscillation or slow speed of convergence. In this paper, directly using energy efficiency as the reward function causes the reward value too large and it is easy to fall into the local optimum. As shown in our experimental results, our designed reward function works well.

(2) The TQL is proposed by combining traditional Q-Learning and multistage decision process which has a loop iteration structure, each sub-Q-Learning solving each sub-problem which is from an original optimization problem. It effectively deals with the dimensional explosion problem caused by the action space increasing in RL and greatly reduces the complexity of optimization problems.

(3) The relevant parameters of each sub-problem can be adjusted independently. Thus, the complexity is low in our proposed TQL algorithm. Simulation results show that the TQL algorithm can solve the original problem with efficiency and flexibility.

The rest of the paper is organized as follows. Related works are summarized in Section 2. Section 3 introduces the system model. In Section 4, the energy efficiency model is formulated. Our proposed algorithm is presented in Section 5. Section 6 shows the simulation framework and numerical results, and conclusions are drawn in Section 7.

## 2. Related Works

According to the method of solving the problem of resource optimization in HetNets, related works are mainly classified into three aspects as traditional optimization methods, machine learning based approaches, and neural-network-based ones.

### 2.1. Traditional Methods for Optimizing Hetnets

For a cluster sleeping method, Chang et al. [11] utilized a genetic algorithm to achieve dynamic matching of energy consumption and Li et al. [12] proposed a Gauss–Seidel method to optimize resources in HetNets. In [13], a low complexity algorithm based on the many-to-many matching game between the virtual SMSs, and the users were proposed to solve the problems of exponential growth of mobile data traffic and energy saving. Anany et al. [14] also utilized matching game and proposed an association algorithm which jointly considered the rate and power of each wireless device to get optimal association between the wireless devices and the best BSs according to a well-designed utility function. Wang et al. [15] modeled the location of each layer of BS as an independent Poisson Point Process (PPP) to analyze the coupling relationship between the probability of successful transmission and BS activation. Dong et al. [16] adopted the Poisson clustering process (PCP) method and analyzed the local delay of discontinuous transmission (DTX) mode. For correctly deploying and expanding HetNets to avoid co-channel interference (CCI), Khan et al. [17] proposed a new three-sector three-layer frequency division multiplexing technology (FFR-3SL). Tiwari et al. proposed a Bayesian minimum mean-square-error (MMSE)-based method to estimate user velocity in [18] and presented a handover-count based minimum-variance-unbiased [19].

### 2.2. Machine Learning for Optimizing Hetnets

Chang et al. [20] utilized a dynamic programming method to optimize spectrum resources between eNBs and low power consumption nodes (LPNs). Deb et al. [21] presented a measurement data-driven machine learning mode LeAP for power control of LTE uplink interference. Chen et al. [22] put forward a method based on hypergraph clustering to solve the serious accumulated interference and improve the system throughput under the requirement of users’ fairness. Different from [8,9], Siddavaatam et al. [23] investigated an energy-aware algorithm based on ant colony optimization. Huang et al. [24] proposed an algorithm based on cross entropy (CE) by a sampling approach to address the problem of user association in an iterative mechanism. Castro-Hernandez et al. [25] proposed the application of clustering algorithm and data mining technology to identify the edge users. Castro-Hernandez et al. [26] also presented clustering algorithm and data mining technology to allow the BS to learn and recognized the received signal strength value autonomously which was from the users’ reports in the handover (HO) process. Like [26], according to the trigger condition of user handoffs in BS, Yao et al. [27] proposed that the minimum numbers of user handoffs was transformed into the volume of transmitted data in a certain period of time as a reward function, and then the historical information for volume of transmitted data was used to approximate expectation for the volume of transmitted data by Monte Carlo algorithm. Different from the action based on our proposed scheme, Fan et al. [28] proposed to decompose the QL based on state composed of user-BS into two QLs based on the state of user and BS.

### 2.3. Neural Networks for Optimizing Hetnets

Many researchers focused on solving heterogeneous network problems with neural networks. Different from Refs. [4,5], Li et al. [29] use convolutional neural networks (CNN) and deep neural network (DNN) network structure as actor part and critic part of the AC algorithm to optimize heterogeneous network resources. Chai et al. [30] proposed an access network modeling and an adaptive parameter adjustment algorithm based on a neural network model. The algorithm was applied to the source and destination switching networks, and the input parameters of users in HetNets were dynamically adjusted according to the required QoS. Fan et al. [31] proposed a fuzzy neural network based on RL to optimize antenna tilt and power to achieve automatic collaborative optimization of power and antenna tilt. Self organizing network entities used cooperative Q-learning and reinforcement back-propagation methods to obtain and adjust their optimization experience to achieve cooperative learning. Similar to [29], some schemes combining neural network with reinforcement learning ware proposed in [32,33]. Since traditional iterative optimization methods, whether optimal or heuristic, usually needed a lot of iterations to achieve satisfactory performance, and led to considerable computational delay, from the perspective of in-depth learning, Lei et al. [32] proposed a feasible cache optimization method which was to train the optimization algorithms through a DNN in advance, instead of directly applying them in real-time caching or scheduling. The computational burden was transferred to a DDN training phase to reduce the complexity of delay sensitive operation phase significantly. Considering the complexity of base station power optimization in multi-layer heterogeneous networks, Sun et al. [33] proposed a dynamic pico-cell base stations (PBS) operation scheme based on CNN, which dynamically changed the on/off state of PBS according to the user’s real-time position, thus reducing the total power of BSS.

As shown in the above analysis, with the increasing complexity of heterogeneous network, more and more parameters are needed to be jointly considered to optimize the network system. It is more difficult to directly solve the network optimization problem by using the traditional optimization scheme. Recently, machine learning technologies have become popular and are applied to the optimization of heterogeneous network resources. Model-free learning brings the convenience of solving non convex problems. However, the acquisition of samples, the design of data labels, and the establishment of Markov decision-making process are all great challenges. In this paper, our proposed TQL algorithm which combines Model-free QL with multistage decision process to optimize the allocation of network resources.

## 3. System Model

**Figure 1:**
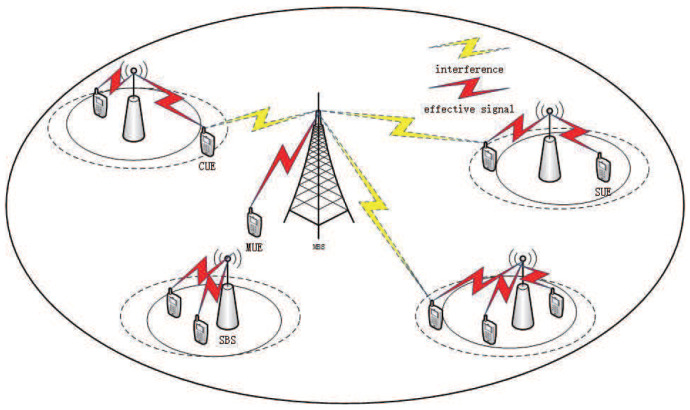
HetNets system model.

We consider a two-layer HetNets scenario as shown in Figure 1, in which a cell contains the macro base stations (MBSs) and SBSs. The SBSs are randomly deployed within the coverage of MBSs. The sets of the SBSs and the MBSs are denoted as *S* and *M*, respectively. The users (UEs) randomly enter the cell. According to a set of UEs association with BSs, UEs are divided into SBS UEs (SUEs) and MBS UEs (MUEs) who are associated with SBSs and MBS, respectively.

In order to balance the load of the entire system network and reduce cross-layer interference by offloading the users of MBSs to SBSs, the enhanced Inter-cell Interference Coordination (eICIC) technology was proposed with two important parameters as ABS and CRE according to [34]. To reduce signal interference, MBSs and SBSs use radio resources in different time periods (subframes) according to eICIC. A frame is divided into some sub frames as ABS and non-ABS subframes, and MBSs normally transmit normal power in nABS (non-ABS) subframes and keep silent or transmit low power in ABS subframes, where the ratio of ABS in a frame is donated as *α*. The SBSs keep normal transmit power in the whole frames. In the time domain, since the MBSs are allowed to be muted in an ABS subframe period, the interference of the MBSs to the users serviced by SBSs is reduced. Therefore, the SINR of UEs with poor channel condition is improved since there is no interference from MBSs in these ABS subframes.

In general, the power of MBSs is much higher than that of SBSs, and some UEs should be accessed to the MBSs according to the reference signal receiving power (RSRP). This is because the UEs in LTE networks are associated with the BSs based on RSRP policy where the UEs are connected to the BSs with the highest reference signal. To balance load and improve the system capacity, SBSs are designed to enhance the frequency multiplexing of the network. CRE was proposed to support SBSs to extend their coverage by adding a bias to their RSRP in which users outside the edge of the SBSs can be connected to the SBSs. The UEs located in the extended area of the SBSs receive less interference from MBSs in ABS subframes and get better channel gain to improve their SINR.

Due to the two operating modes of the MBSs in the ABS subframes and non-ABS subframes (nABS), there are also two interference modes for the UEs in the downlink in the HetNets. When the MBSs are in the ABS subframes, the UEs receive only the signal transmitted by the SBSs. However, the MBSs are in the nABS subframes, and the UEs are interfered by the transmitted signal from the SBSs and the MBSs.

The $SINR_{k,n}$ of the UE *n* connected to the MBS *k* can be expressed as

(1) $$\begin{array}{c} SINR_{k,n}=\left\{\begin{array}{cc} \frac{P_{M}^{k,n}G_{k,n}}{\sum_{j\in M,j\neq k}P_{M}^{j,n}G_{j,n}+\sum_{i\in S}P_{S}^{i,n}G_{i,n}+N_{0}} & k\in M^{nABS}\\ \frac{P_{m}^{k,n}G_{k,n}}{\sum_{j\in M,j\neq k}P_{m}^{j,n}G_{j,n}+\sum_{i\in S}P_{S}^{i,n}G_{i,n}+N_{0}} & k\in M^{ABS} \end{array}\right. \end{array}$$

where $P_{M}^{k,n}$ is the transmission power from the MBS *k* to the UE *n* in the nABS subframes, $P_{m}^{j,n}$ is the transmission power from the MBS *j* to the UE *n* in the ABS subframes, $G_{k,n}$ represents the channel gain from the MBS *k* to UE *n*, $P_{S}^{j,n}$ denotes the transmission power from the SBS *j* to the UE *n*, $N_{0}$ indicates noise variance of the additive white Gaussian, $M^{ABS}$ and $M^{nABS}$ are denoted as MBSs in the ABS and nABS subframes periods, respectively. Note that *m* and *M* are short for $M^{ABS}$ and $M^{nABS}$, respectively.

The $SINR_{k,n}$ of the UE *n* connected in the SBS *k* can be written as

(2) $$SINR_{k,n}=\left\{\begin{array}{cc} \frac{P_{S}^{k,n}G_{k,n}}{\sum_{j\in M,j=k}P_{M}^{j,n}G_{j,n}+\sum_{i\in S,i\neq k}P_{S}^{i,n}G_{i,n}+N_{0}} & k\in S^{nABS}\\ \frac{P_{S}^{k,n}G_{k,n}}{\sum_{j\in m,j=k}P_{m}^{j,n}G_{j,n}+\sum_{i\in S,i\neq k}P_{S}^{i,n}G_{i,n}+N_{0}} & k\in S^{ABS} \end{array}\right.$$

where $S^{ABS}$ and $S^{nABS}$ are denoted as the SBSs sets in the ABS subframes and nABS subframes, respectively. Hence, the transmission rate of UE *n* connected to the BS *k* can be given as

(3) $$R_{k,n}=\left\{\begin{array}{cc} \left(1-\alpha \right)B\log\left(1+SINR_{k,n}\right) & k\in M^{nABS}\cup S^{nABS}\\ \alpha B\log\left(1+SINR_{k,n}\right) & k\in M^{ABS}\cup S^{ABS} \end{array}\right.$$

where *B* is the system bandwidth.

## 4. Problem Formulation

For comprehensively optimizing the energy efficiency of HetNets, the parameters such as SI-SBSs, ABS, and CRE should be jointly considered. The optimization problem is modeled by setting the energy efficiency of the system as the optimization objective function. Based on the above analysis, we can establish a joint optimization energy efficiency problem as

(4) maxxk,n,αPk∑k∈M∪S∑u∈Uxk,nlogRk,n∑k∈M∪SPks.t.0≤xk,n(1)∼(3)

where the relationship during state $x_{k,n}$, the CRE setting size of the SB *k* and transmission power $P_{k,n}$ is closely related. $\sum_{k\in M\cup S}P_{k}$ is closely related to the number of active SBSs.

Let $x_{k,n}$ represent the connection status between BS *k* and UE *n*, which is expressed as

(5) $$x_{k,n}=\left\{0,1\right\}\;\forall k\in M\cup S,n\in U$$

(6) $$\;\sum_{k\in M\cup S}x_{k,n}=1\;\forall n\in U$$

where $x_{k,n}=1$ indicates that a connection is established between BS *k* and UE *n*, otherwise 0. Equation (6) represents that each UE in the cell can only be connected to one BS.

The transmission power from the BS *k* to the UE *n* at different subframe times can be expressed as

(7) $$\begin{array}{c} P_{k,n}=\left\{\begin{array}{cc} P_{M}^{k,n} & \;k\in M^{nABS}\\ P_{m}^{k,n} & \;k\in M^{ABS}\\ P_{{}_{S}}^{k,n} & \;k\in S \end{array}\right. \end{array}$$

where

(8) $$P_{M}^{k,n}=\cdot N_{\;T\;R\;X}\cdot \left(P_{0}^{m}+R^{k}\cdot P_{{}_{\max}}^{m}\right)$$

(9) $$P_{m}^{k,n}=N_{\;T\;R\;X}\cdot P_{0}^{m}$$

where $N_{\;T\;R\;X}$ is the number of BS transceivers, $P_{0}^{m}$ indicates MBSs consumption power in sleep state, and $P_{{}_{\max}}^{m}$ represents maximum transmission power of the MBSs, $R^{k}\in \left[0,1\right]$ denotes the load factor of the BSs, which depends on ABS, CRE, and the load density of the BSs, and $P_{S}^{k,n}$ is

(10) $$P_{S}^{k,n}=e^{k}\cdot N_{\;T\;R\;X}\cdot \left(P_{0}^{s}+R^{k}\cdot P_{\max}^{s}\right)+\left(1-e^{k}\right)\cdot N_{\;T\;R\;X}\cdot P_{sleep}^{s}+\Delta$$

where $e^{k}$ represents active state which is 1, and otherwise 0, $P_{\max}^{s}$ denotes the maximum RF output power of the SBSs, and $P_{0}^{s}$ indicates the power when there is no RF of SBSs, $P_{sleep}^{s}$ is the power consumption when the BSs transceiver station are in sleep state, and Δ is

(11) $$\Delta =\left\{\begin{array}{cc} \varphi \cdot P_{0} & \mathrm{When}\;\mathrm{the}\;\mathrm{base}\;\mathrm{station}\;k\;\mathrm{switches}\;\mathrm{from}\\ & \mathrm{the}\;\mathrm{sleep}\;\mathrm{state}\;\mathrm{to}\;\mathrm{the}\;\mathrm{active}\;\mathrm{state}.\\ 0 & \mathrm{Others} \end{array}\right.$$

where φ represents the proportion of the BSs that wake up the transceiver from sleep to activation state.

Note that ABS, CRE, and the number of active SBSs all affect the load factor of the BSs, which makes problem (4) become complicated and be a non-convex problem. In order to fully consider the complexity and unknown characteristics of the real environment, the optimization problem (4) can be changed as

(12) maxα,βSfα,β,S=maxxk,n,αPk∑k∈M∪S∑u∈Uxk,nlogRk,n∑k∈M∪SPks.t0≤xk,n(1)∼(11)

where *f* function is unknown, β represents the CRE parameter, $\left|S\right|$ represents the number of SBSs activations.

## 5. Solution with a Tql Algorithm

It is difficult to solve (12) directly because the complexity of the target optimization system is an unknown non-convex problem. In [12], the Gauss–Seidel method needs too much prior knowledge, which is not as convenient as reinforcement learning. In this paper, the table reinforcement learning method QL is used to optimize the system energy efficiency and then our TQL algorithm is proposed to optimize it.

### 5.1. Q-Learning Algorithm

The environment is typically formulated as a finite-state Markov Decision Process (MDP) and we set a finite discrete time series $t\in \left\{0,1,\ldots ,\infty \right\}$. ABS and CRE are denoted by α∈A and β∈B, respectively. The activation state of the SBSs in the state $u_{t}$ is $e_{t}\in E$, where $E={\left(0,1\right)}^{\left|P\right|}$, and $\left|P\right|$ represents the number of SBSs in a cell. According to the Control Space Augmentors (CSA) concept mentioned in [9], the SBSs states $e_{t}$ can be derived based on the number of SBSs activations $\left|S\right|$ in the cell, where A, B, and $\left|S\right|$ represent a limited set of all parameter configurations. Let S be a discrete set of environment states and A be a discrete set of actions. At each step *t*, the agent senses the environment state $s_{t}=s\in S$ and selects an action $a_{t}=a\in A$ to be performed, where *s* is the position of a certain number of UEs in the cell, S represents the set of cell UEs positions, *a* is optimal α, β and $\left|S\right|$ parameter configurations to optimize the energy efficiency of the system, and A represents the set of parameter configurations. As a result, the environment makes a transition to the new state $s_{t+1}=s^{\prime }\in S$ according to probability $P(s_{t+1}=s{}^{\prime }|s_{t}=s,a_{t}=a)$ and thereby generates a reward $r_{t}=r\left(s_{t},a\right)\in R$ passing to the agent. MDP is denoted as a tople (S,A,P,R), where

• S is the set of finite state space;
• A is the set of finite action space;
• P is the set of transition probabilities;
• R represents the set of reward funtion.

(1) State: The position of users at step *t* is considered as state $s_{t}=s$, and the set of states is denoted as S.

(2) Action: The ABS configuration α, the CRE bias β, and the number of SBS activations $\left|S\right|$ are considered as action $a_{t}=\left(\alpha ,\beta ,\left|S\right|\right)$ at state $s_{t}$ where α∈A, β∈B and $\left|S\right|\in \left\{0,1,\ldots ,\left|P\right|\right\}$. The action space size is $X=\left(\left|P\right|+1\right)\times \left|A\right|\times \left|B\right|$.

(3) State transition: The location of users in the cell changes is considered irregularly, and the state transition is random.

(4) Reward function: The optimization problem is system energy efficiency which is used as reward function, but, in the actual simulation process, the reward is too large, which causes the system to fall into the local optimum easily. Our proposed solution is that negative reciprocal of energy efficiency is designed as the reward.

The goal of RL is to find out the expectation of the strategy with the greatest cumulative reward, which can be expressed as

(13) $$\begin{array}{c} \max_{\pi }E\left[\sum_{t=0}^{\infty }\gamma^{t}r\left(s_{t},a_{t},s_{t+1}\right)\right] \end{array}$$

where discount factor γ indicates the degree of influence of successor states on current state, and $r\left(s_{t},a_{t},s_{t+1}\right)$ represents the reward of state $s_{t}$ selecting $a_{t}$ and then transiting to state $s_{t+1}$.

The best decision sequence of MAP is solved by the Bellman equation. The state-action value function q(s,a) can evaluate the current state. The value of each state-action is not only determined by the current state but also by the successor states. Therefore, the state-action value function q(s,a) of the current s can be obtained by the cumulative reward expectation of the state. Bellman’s equation can be given as [35]

(14) $$\begin{array}{c} q_{\pi }\left(s,a\right)=E_{\pi }\left[r_{t+1}+\gamma r_{t+2}+\gamma^{2}r_{t+3}+\ldots |a_{t}=a,s_{t}=s\right], \end{array}$$

which is also equivalent to

(15) $$\begin{array}{c} q_{\pi }\left(s,a\right)=E_{\pi }\left[r_{t+1}+\gamma q_{\pi }\left(s_{t+1},a_{t+1}\right)|a_{t}=a,s_{t}=s\right] \end{array}$$

Optimal action-value function $Q^{*}\left(s,a\right)=\max_{\pi }Q^{*}\left(s,a\right)$ can be written as

(16) $$\begin{array}{c} Q^{*}\left(s,a\right)=\sum_{s^{{}^{\prime }}}P(s^{{}^{\prime }}|s,a)\left(r\left(s,a,s^{{}^{\prime }}\right)+\gamma \max_{a^{{}^{\prime }}}Q^{*}\left(s^{{}^{\prime }},a^{{}^{\prime }}\right)\right) \end{array}$$

The update process of Q-value using a time difference method is expressed as [35]

(17) $$\begin{array}{c} Q\left(s,a\right)\leftarrow Q\left(s,a\right)+\lambda \left[r+\gamma \max_{a^{{}^{\prime }}}Q\left(s^{{}^{\prime }},a^{{}^{\prime }}\right)-Q\left(s,a\right)\right] \end{array}$$

where λ is the learning rate. According to the Formula (17), the QL algorithm is utilized to solve problem (12) as shown in Algorithm 1:
**Algorithm 1:** The QL for optimizing original problem. **Require:** the set of state *K*, the set of action *X*, earning rate $\lambda_{\mathrm{QL}}$, greedy probability $\varepsilon_{\mathrm{QL}}$,  discount factor $\gamma_{\mathrm{QL}}$, and $threshold_{\mathrm{QL}}$. **Ensure:** Q table.  1: Initialize Q(s,a), state *s* and *n* = 0 and setting $threshold_{\mathrm{QL}}$;  2: **while**
*n* <= $threshold_{\mathrm{QL}}$
**do**  3:  In state *s*, select the optimal action *a* with greedy probability $\varepsilon_{\mathrm{QL}}$;  4:  Observe *r*;  5:  randomly transfer from *s* to $s^{{}^{\prime }}$;  6:  Update Q(s,a) according to Formula (17);  7:  $s\leftarrow s^{{}^{\prime }}$;  8:  n=n+1;  9: **end while**  10: Output: Q table;

### 5.2. Tql Algorithm

If QL is directly utilized to solve the original problem (12) as shown in Algorithm 1, we can see the action size $X=\left(\left|P\right|+1\right)\times \left|A\right|\times \left|B\right|$ is too large, where $\left|•\right|$ represents a cardinality of set. Since the action is represented by a vector of three dimensions, the optimization problem can be decomposed into three subproblems. We propose the TQL algorithm which decomposes the objective optimization problem into three sub-problems as optimizing the ratio of ABS α, CRE bias β, and the number of SBS activations $\left|S\right|$ to reduce action space size.

#### 5.2.1. Sub-Problem A: Given the Cre Bias β and the Number of Sbs Activation $\left|S\right|$ for Optimizing the Abs Ratio α

The action of the sub-problem A is $a_{t}=\left(\alpha ,\beta ,\left|S\right|\right)$ where β and $\left|S\right|$ are given. α∈A and the action space size is $\left|A\right|$. State *s* = $s_{t}$. The tabular method of Q-learning can be used to solve sub-problem A and the updating rule of sub-Q-value can be written as

(18) $$\begin{array}{c} Q_{\alpha }\left(s,a\right)\leftarrow Q_{\alpha }\left(s,a\right)+\lambda_{1}\left[r+\gamma_{1}\max_{a^{{}^{\prime }}}Q_{\alpha }\left(s^{{}^{\prime }},a^{{}^{\prime }}\right)-Q_{\alpha }\left(s,a\right)\right] \end{array}$$

and shown in Algorithm 2.
**Algorithm 2:** The CRE bias β and the number of SBSs activation $\left|S\right|$ are given to optimize the ABS ratio α. **Require:**
$A_{t}=\left(\alpha ,\beta ,\left|S\right|\right)$. **Ensure:** Optimized ABS ratio α.  1: Initialize $Q_{\alpha }\left(s,a\right)$, state *s* and *n* = 0;  2: Setting learning rate $\lambda_{1}$, greedy probability $\varepsilon_{1}$, discount factor $\gamma_{1}$ and $threshold_{1}$;  3: **while**
*n* <= $threshold_{1}$
**do**  4:  In state *s*, select the optimal action *a* with greedy probability $\varepsilon_{1}$;  5:  Observe *r*;  6:  randomly transfer from *s* to $s^{{}^{\prime }}$;  7:  Update $Q_{\alpha }\left(s,a\right)$ according to Formula (18);  8:  $s\leftarrow s^{{}^{\prime }}$;  9:  n=n+1;  10: **end while**  11: Output: α=a;

#### 5.2.2. Sub-Problem B: Given the Abs Ratio α and the Number of Sbs Activation $\left|S\right|$ for Optimizing the Cre Bias β

The action of the sub-problem B is $a_{t}=\left(\alpha ,\beta ,\left|S\right|\right)$ where α and $\left|S\right|$ are known. β∈B and the action space size is $\left|B\right|$. State *s* = $s_{t}$. Like Formula (18), the updating rule of sub-Q-value can be written as

(19) $$\begin{array}{c} Q_{\beta }\left(s,a\right)\leftarrow Q_{\beta }\left(s,a\right)+\lambda_{2}\left[r+\gamma_{2}\max_{a^{{}^{\prime }}}Q_{\beta }\left(s^{{}^{\prime }},a^{{}^{\prime }}\right)-Q_{\beta }\left(s,a\right)\right] \end{array}$$

and shown in Algorithm 3.
**Algorithm 3:** The ABS ratio α and the number of SBSs activation $\left|S\right|$ are given to optimize the CRE bias β. **Require:**
$a_{t}=\left(\alpha ,\beta ,\left|S\right|\right)$. **Ensure:** Optimized CRE bias β.  1: Initialize $Q_{\beta }\left(s,a\right)$, state *s* and *n* = 0;  2: Setting learning rate $\lambda_{2}$, greedy probability $\varepsilon_{2}$, discount factor $\gamma_{2}$, and $threshold_{2}$;  3: **while**
*n*<= $threshold_{2}$
**do**
  4:  In state *s*, select the optimal action *a* with greedy probability $\varepsilon_{2}$;  5:  Observe *r*;  6:  randomly transfer from *s* to $s^{{}^{\prime }}$;  7:  Update $Q_{\beta }\left(s,a\right)$ according to Formula (19);  8:  $s\leftarrow s^{{}^{\prime }}$;  9:  n=n+1;  10: **end while**  11: Output: β=a;

#### 5.2.3. Sub-Problem C: Given the Abs Ratio α and the Cre Bias β for Optimizing the Number of Sbs Activation $\left|S\right|$

The action of the sub-problem B is $a_{t}=\left(\alpha ,\beta ,\left|S\right|\right)$ where α and β are known. $\left|S\right|\in \left\{0,1,\ldots ,\left|P\right|\right\}$ and the action space size is $\left|P\right|+1$. State *s* = $s_{t}$. It is similar to Formula (18) and the updating rule of sub-Q-value can be written as

(20) $$\begin{array}{c} Q_{\left|S\right|}\left(s,a\right)\leftarrow Q_{\left|S\right|}\left(s,a\right)+\lambda_{3}\left[r+\gamma_{3}\max_{a^{{}^{\prime }}}Q_{\left|S\right|}\left(s^{{}^{\prime }},a^{{}^{\prime }}\right)-Q_{\left|S\right|}\left(s,a\right)\right] \end{array}$$

and shown in Algorithm 4.
**Algorithm 4:** The ABS ratio α and the CRE bias β are given to optimize the number of SBS activation $\left|S\right|$. **Require:**
$a_{t}=\left(\alpha ,\beta ,\left|S\right|\right)$. **Ensure:** Optimized ABS ratio α.  1: Initialize $Q_{\left|S\right|}\left(s,a\right)$, state *s* and *n* = 0;  2: Setting learning rate $\lambda_{3}$, greedy probability $\varepsilon_{3}$, discount factor $\gamma_{3}$, and $threshold_{3}$  3: **while**
*n* <= $threshold_{3}$
**do**  4:  In state *s*, select the optimal action *a* with greedy probability $\varepsilon_{3}$;  5:  Observe *r*;  6:  randomly transfer from *s* to $s^{{}^{\prime }}$;  7:  Update $Q_{\left|S\right|}\left(s,a\right)$ according to Formula (20);  8:  $s\leftarrow s^{{}^{\prime }}$;  9:  n=n+1;  10: **end while**  11: Output: $\left|S\right|=a$;

The TQL algorithm solves the original problem (11) shown in Algorithm 5.
**Algorithm 5:** The algorithm for optimizing initial problems. **Require:**
α∈A, β∈B, $\left|S\right|=\left\{0,1,\ldots ,{\left|S\right|}_{\max}\right\}$, Reward *r*, Learning rate $\lambda =\left\{\lambda_{1},\lambda_{2},\lambda_{3}\right\}$,  Greedy probability $\varepsilon =\left\{\varepsilon_{1},\varepsilon_{2},\varepsilon_{3}\right\}$ and Discount factor $\gamma =\left\{\gamma_{1},\gamma_{2},\gamma_{3}\right\}$. **Ensure:** Optimal action configuration $\left\{\alpha ,\beta ,\left|S\right|\right\}$ in each state.  1: Initialize $U_{t}$, α, β, $\left|S\right|$,  2: **while**
*n* <= $threshold_{4}$
**do**  3:  Fixed the CRE bias β and the number of SBS activation $\left|S\right|$, calculate the ABS ratio α   according to Algorithm 2. Pass the solved α to step (4) and step (5);  4:  Fixing the ABS ratio α and the number of SBS activation $\left|S\right|$, calculate the CRE bias β   according to Algorithm 3. Pass the solved β to step (3) and step (4);  5:  Fix the ABS ratio α and the CRE bias β, calculate the number of SBS activation $\left|S\right|$   according to Algorithm 4. Pass the solved $\left|S\right|$ to step (4) and step (3);  6:  n=n+1;  7: **end while**  8: Output: α, β, $\left|S\right|$;

In summary, our scheme has changed the size of action space from traditional exponential increase as $X=\left(\left|P\right|+1\right)\times \left|A\right|\times \left|B\right|$ to linear increase as $X=\left(\left|P\right|+1\right)+\left|A\right|+\left|B\right|$, which greatly reduces the dimension and size of the action space.

**Figure 2:**
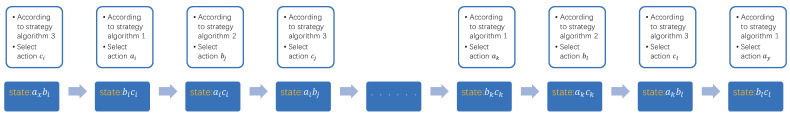
Multi-stage decision process.

Algorithm 5 can be considered as a multi-stage decision process optimization problem which is shown in Figure 2. The action spaces of the third, fourth, and fifth step in Algorithm 5, which are the optimization problem of Algorithms 2–4, are set to A, B, and C where A, B, C are limited and denoted as set *A*, *B*, and $\left\{0,1,\ldots ,\left|P\right|\right\}$, respectively. It can be seen in Figure 2 that the state spaces of the third, fourth, and fifth step in Algorithm 5 are the Cartesian product of the other two action spaces and the state spaces size are $\left|B\right|\left|C\right|$, $\left|A\right|\left|C\right|$ and $\left|A\right|\left|B\right|$, respectively. The state transition equation refers to the transition probability from state $b_{i}c_{i}$ to state $a_{i}b_{i}$ conditioned on taking action $a_{i}$. The state transition probability is written as $P\left(s^{{}^{\prime }}=a_{i}b_{i}|s=b_{i}c_{i},action=a_{i}\right)=1$, where $a_{i}\in A$, $b_{i}\in B$ and $c_{i}\in C$. We assume with the condition that there is no interference in the transition process, so the transition probability here is 1. If the multi-stage decision-making process is a closed loop that $b_{i}c_{i}=b_{k}c_{k}$ or $a_{i}c_{i}=a_{k}c_{k}$ or $a_{i}b_{j}=a_{k}b_{l}$ exists, Algorithm 5 is stable. Since $\left|A\right|$, $\left|B\right|$, and $\left|C\right|$ are all bounded, $b_{i}c_{i}=b_{k}c_{k}$ or $a_{i}c_{i}=a_{k}c_{k}$ or $a_{i}b_{j}=a_{k}b_{l}$ appears at most $\min\left(\left|B\right|\left|C\right|,\left|A\right|\left|C\right|,\left|A\right|\left|B\right|\right)+1$ transitions during the whole stage. Therefore, it indicates that Algorithm 5 is stable. In Section 6, Figure 6 further illustrates that Algorithm 5 converges to a near optimal solution.

### 5.3. Neural Network for the Classification of States

**Figure 3:**
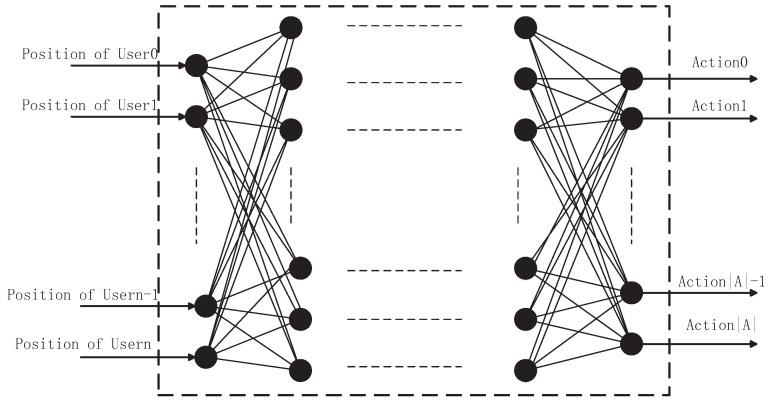
The inputs are the users’ position, labels are the optimal action in each state.

In subsection B, the TQL algorithm solves the problem of action space dimension explosion. In order to have the ability to classify the state for the agent, a DNN whose structure is shown in Figure 3, in which there are two hidden layers and each hidden layer has 512 nodes. The activation function for each hidden layer is a rectified linear unit (ReLU), and ADAM (adaptive moment estimation) [36] is utilized as updating algorithm and learning-rate is equal to 0.001. The optimal strategy of TQL is to label the samples and specify optimal action in each state. Samples are the set of users position which are gathered from TQL algorithm. The input is the location information of users and the optimal action is encoded according to the index in the action space as the output.

We tried a one-layer, two-layer, and three-layer hidden layer network, and the experimental results showed that it had similar performance on the training performance. Two hidden layers DNN performed relatively more effective with respect to training speed and performance, which is why we use two hidden layers of DNN.

## 6. Numerical Simulation

**Figure 4:**
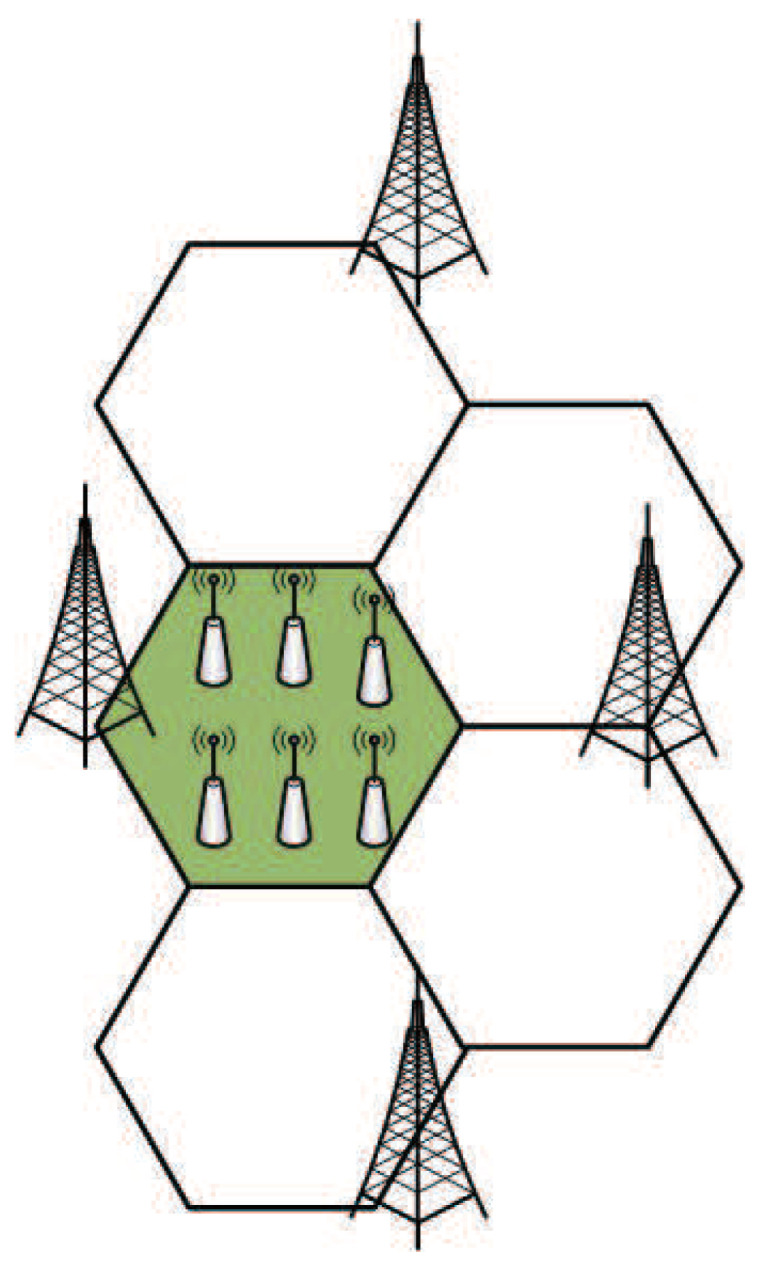
Simulation scenario.

The parameters of experimental simulation are set according to the 3GPP LTE-A HetNets framework [37], and the wireless channel is modeled as deterministic path loss attenuation and random shadow fading models. In this part, the scenario we deployed is that each MBS covers the users in a 120° cell as shown as shadow part in Figure 4 and is interfered by three other MBSs. We deployed a field where six SBSs are randomly deployed within the coverage of the MBS in the green shaded part of Figure 4 and select working mode according to load conditions.

The coverage radius of the MBS and the SBSs are 500 m and 100 m, respectively. The thermal noise power is −176 dBm, the system spectrum bandwidth is 10 MHz and the antenna gains of the MBSs, SBSs, and the UE are 14 dBi, 5 dBi, and 0 dBi, respectively. The maximum transmission power of the MBSs and the SBSs are set to 46 dBm and 30 dBm, respectively. The probability of a user entering a cell to access a MBS and a SBS are 1/3 and 2/3, respectively. The proportion φ of the BSs that wake up is set here to 0.5. Although LTE frame includes 10 subframes, the ABS mode has a periodicity of eight subframes. The ABS ratio of protected subframe to traditional subframe $\alpha \in \left[0,1\right]$ belongs to the set $\left\{0/8,...,7/8\right\}$. CRE is denoted by β∈B, where $B=\left\{0,6,9,12,15,18\right\}$. The specific parameters are listed in Table 1.

**Figure 5:**
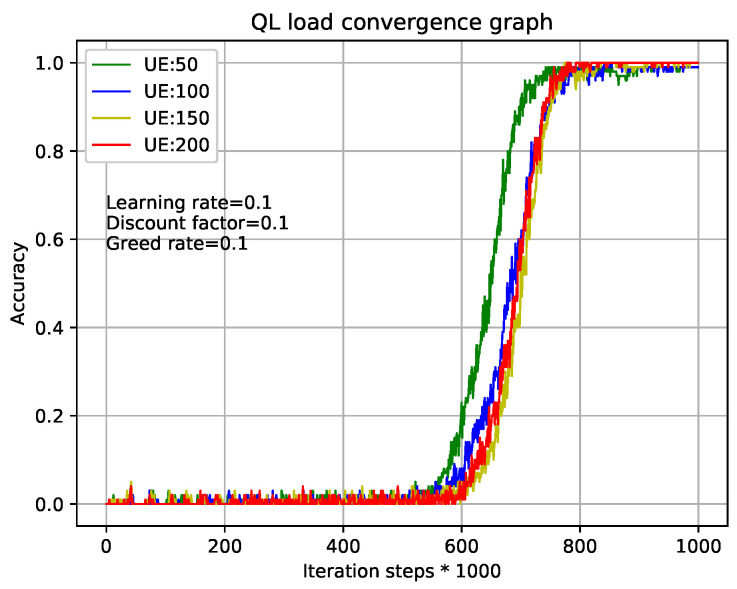
QL algorithm convergence graph with different loads.

**Figure 6:**
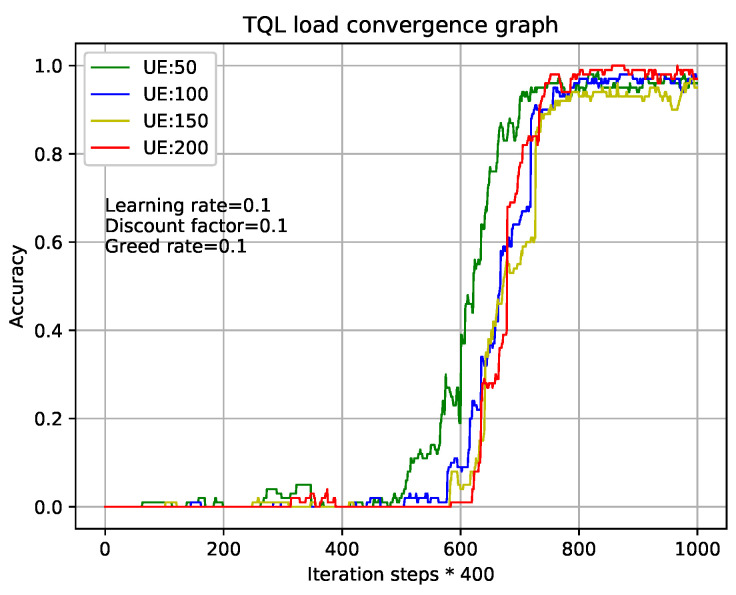
TQL algorithm convergence graph with different loads.

Figure 5 and Figure 6 show the relationship during iterations and accuracy of Q-Learning algorithm and our TQL algorithm where learning rate, discount factor, and greed rate are all set to 0.1, and the number of users are set to 50, 100, 150, and 200, respectively. Figure 5 shows that the tabular method of the Q-Learning algorithm converges after 80×1000= 800,000 iterations under different load conditions. Our proposed TQL algorithm converges after 800×400= 320,000 iterations as shown in Figure 6. We can see that the convergence speed of our proposed TQL algorithm is increased by about 60% compared with the Algorithm 1. Note that the convergence speed of TQL algorithm proposed in this paper is still much faster than that of Algorithm 1, especially in the case where the action space cardinality is very large from the analysis of Algorithm 5.

**Figure 7:**
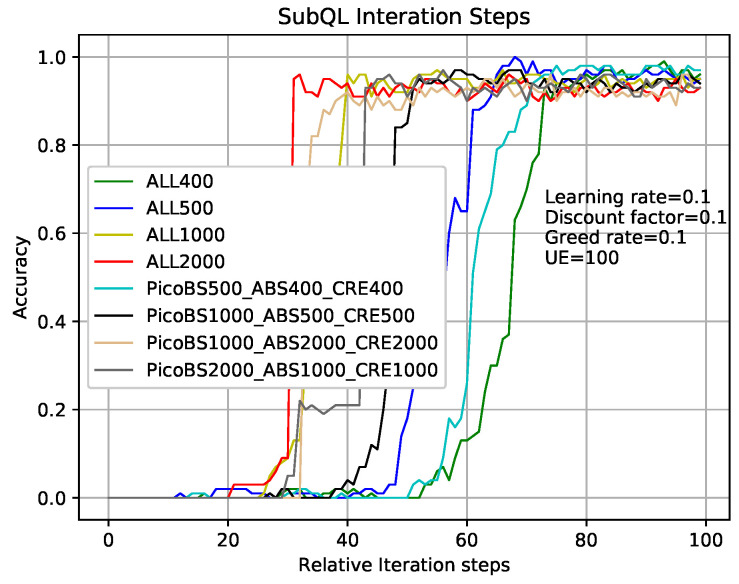
TQL with different sub-QL iteration numbers convergence graph.

Figure 7 shows that the comparison of sub-QLs (Algorithms 2–4) iterations in the TQL algorithm give different results where relative parameters are the same as that in analysis of Figure 5 and the number of users is 100. Take the greed line and red line as examples which indicate the number of sub-QL iterations of SBSs ($threshold_{3}$ in Algorithm 4), ABS ($threshold_{1}$ in Algorithm 2), and CRE ($threshold_{2}$ in Algorithm 3) are all set to 400 and 2000, respectively. When the iteration number of TQL represented by the green line and red line is more than about 75 and 30, our proposed TQL algorithm is convergent, but the final accuracy rates are about 98% and 90%, respectively. We can see that, although the convergence speed respected by green line is relatively slower, the final correct rate is higher and the performance is better than that respected by the red line. Our proposed TQL algorithm can make a balance between performance and convergence speed according to actual requirements by adjusting the iterations of the subsystems such as the black line indicated case where the numbers of sub-QL iterations of SBSs, ABS, and CRE are set to 1000, 500, and 500, respectively. The number of iterations for TQL represented by cyan line is more than about 50 and the final correct rate is about 93%.

**Figure 8:**
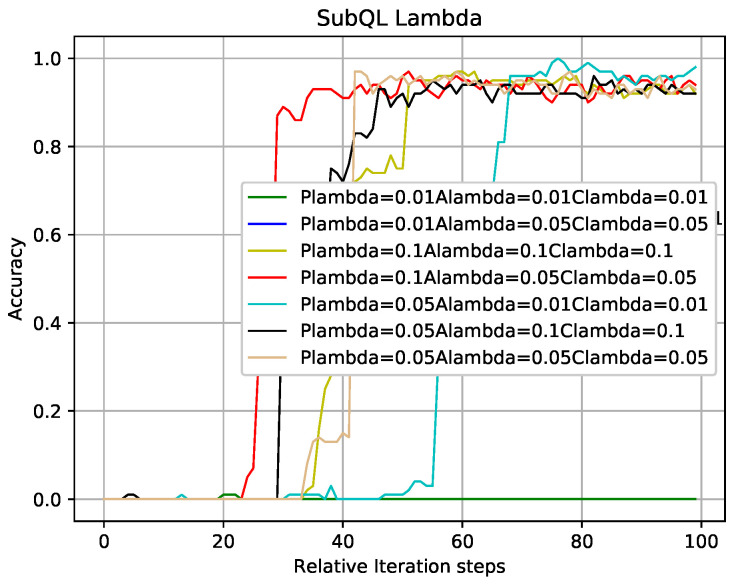
TQL algorithms convergence graph with different learning rates.

In the case where other experimental parameters are the same as that of Figure 7, Figure 8 shows the influence of different learning rates of sub-QL algorithms on convergence speed and correct rate. Take the red line and the cyan line as examples which indicate that the learning rates of sub-QL of SBSs, ABS, and CRE are set to 0.1, 0.05, 0.05 and 0.05, 0.01, 0.01, respectively. We can see that, when the iteration numbers of TQL respected by the red line and cyan line are about 30 and 70, our TQL algorithm is convergent and the final accuracy rate are about 95% and 99%, respectively. The convergence speed respected by the red line is faster than that respected by the cyan line, but the final accuracy rate is otherwise. Pay attention to the problem caused by the setting learning rates respected by the green line which are all set to 0.01, if the learning rate is set too low, the system falls into the local optimum, and the global optimum cannot be found. It is easy to make this mistake in the RL.

**Figure 9:**
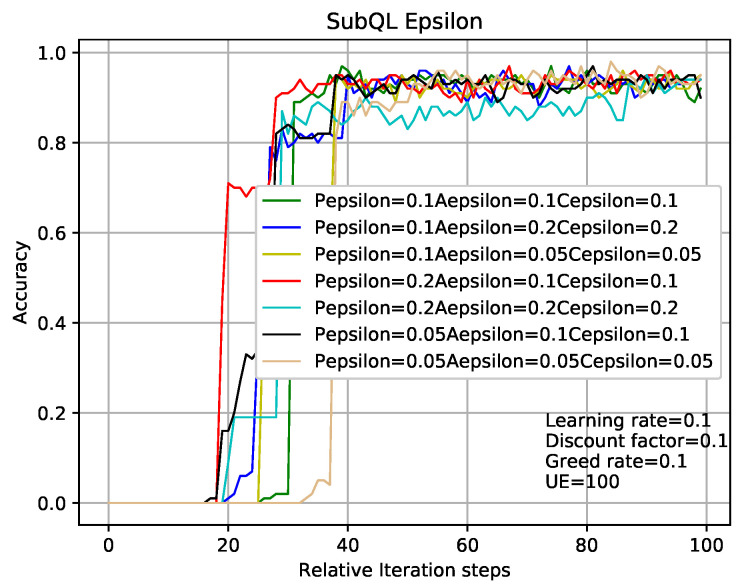
TQL algorithms convergence graph with different greedy rates.

**Figure 10:**
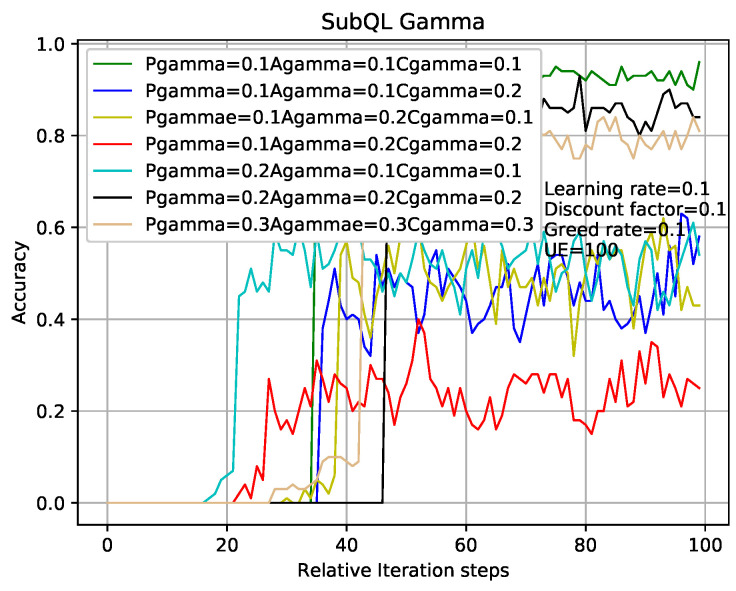
TQL algorithms’ convergence graph with different discount factors.

In the case where other experimental parameters the same as that in analysis of Figure 7 and Figure 8, the methods of analyzing Figure 9 and Figure 10 are like that in Figure 7 and Figure 8. The results obtained from Figure 7, Figure 8, Figure 9 and Figure 10 are that the balance can be obtained between performance and convergence speed by changing the corresponding parameters of the sub-QL in our TQL algorithm. It can be seen that our TQL algorithm has greater flexibility in parameter adjustment compared to the general system where only one set of parameters is set.

**Figure 11:**
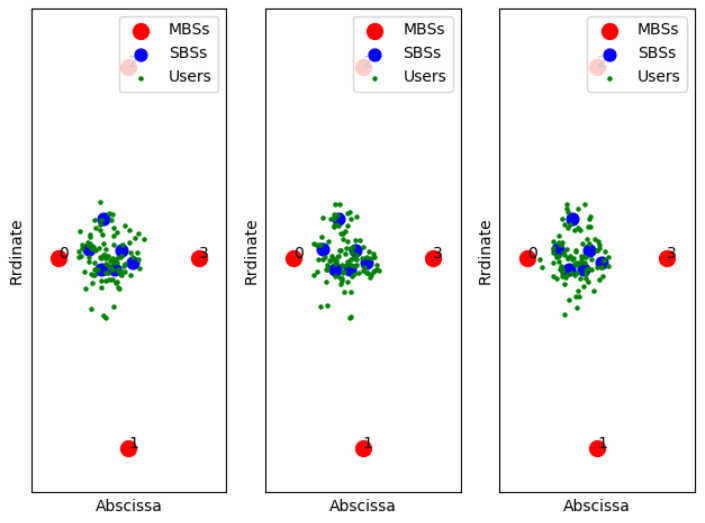
ABS = 0, CRE = 0.

**Figure 12:**
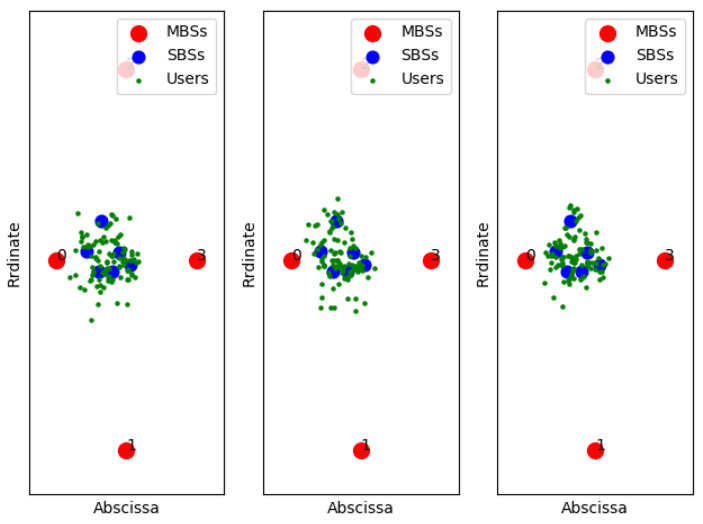
ABS = 3/8, CRE = 6.

**Figure 13:**
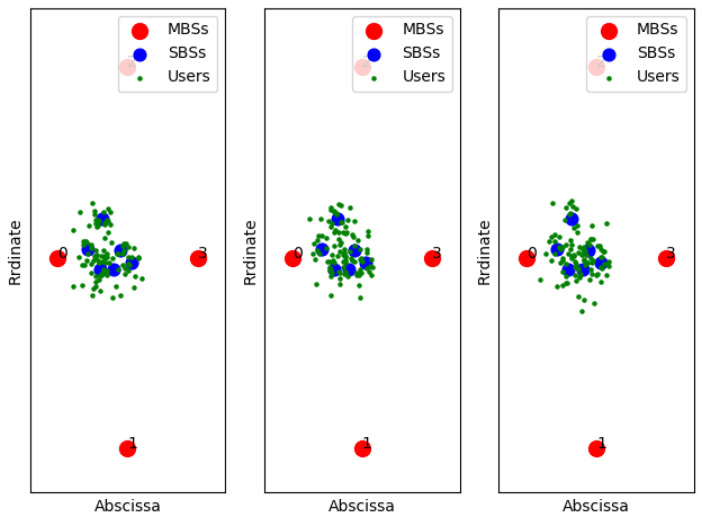
ABS = 7/8, CRE = 18.

Figure 11, Figure 12 and Figure 13 show examples of the sample classification of our designed DNN when the ABS, CRE, and the number of SBSs activations are set to (0, 0, 6), (3/8, 6, 6) and (7/8, 18, 6), respectively. The labeled samples are obtained from the optimal strategy of TQL algorithm, in which 90% of them are training samples and the rest are test samples. The red dots represent the macro base stations where the macro base station with the number 0 is the cell signal source, and the macro base stations with the other numbers are the interference signal sources. Blue and green dots indicate the location of SBSs and users in the cell, respectively.

**Figure 14:**
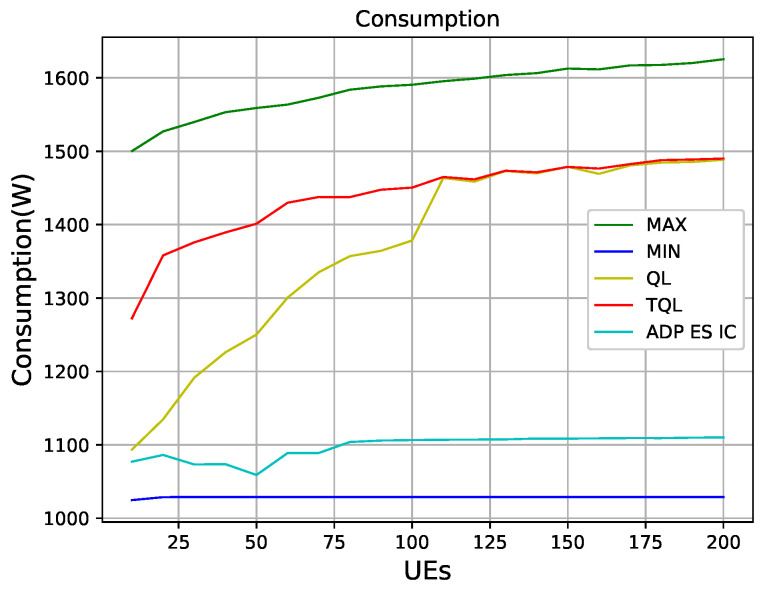
The consumption graphs of different algorithms.

**Figure 15:**
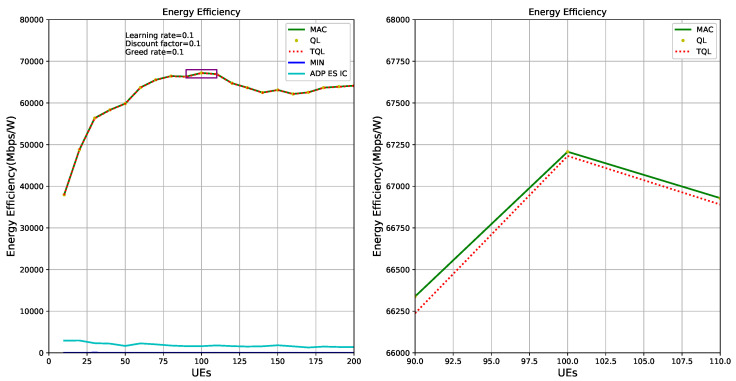
Energy efficiency optimization graphs of different algorithms.

Figure 14 shows that QL, TQL, and the ADP ES IC algorithm in [9] optimize the power consumption of HetNets. We can see that, under the condition that the number of users being less than 100, the power consumption obtained by the QL algorithm is lower than the TQL proposed in this paper. However, when the number of users is greater than 100, the power consumptions of the two algorithms are the same, which are between the maximum power consumption and the minimum power consumption. The algorithm of ADP ES IC optimizes the power consumption best in the case of 50 users, and it is seen in Figure 14 that the power consumption control of the entire system is better than QL and TQL in the entire 10–200 users interval. However, it can be seen that such good results is obtained at the premise of sacrificing energy efficiency in Figure 15.

Figure 15 shows the energy efficiency of HetNets is optimized by QL, TQL, and ADP ES IC algorithms, respectively. Green solid line and red dotted line represent the theoretical optimal energy efficiency and the energy efficiency obtained by our TQL algorithm, respectively. The sub-picture on the left of Figure 15 shows that the optimized energy efficiency of our TQL algorithm is very close to the theoretical optimal, and the sub-picture on the right of Figure 15 shows that the index of energy efficiency of our algorithm optimization system is slightly lower than the theoretical optimal. According to the analysis of Figure 6, because the TQL algorithm has not found the optimal solutions (i.e., Pico BS, ABS, and CRE configuration) in some states, the gap exists between theoretical optimal and TQL algorithm, as shown in the sub-picture on the right of Figure 15. However, Figure 15 proves that the TQL energy efficiency performance is very close to the theoretical optimal energy efficiency, indicating that the TQL algorithm proposed in this paper has not been optimized in some states, but the solution found is also a relatively optimal solution, which may be a suboptimal solution. For the system, the performance loss is small. The ADP ES IC algorithm is poor in energy efficiency optimization, mainly because the authors focus on power optimization of the system in HetNets.

## 7. Conclusions

In order to jointly optimize resources to maximize the energy efficiency of HetNets by RL, there is a problem of too large action space and state space. We propose a novel QL algorithm (TQL) based on the multi-stage decision process to improve the QL algorithm to effectively solve the problem of excessive action space. Through the analysis of Algorithm 5, the advantage is greater in the case of large action space. For the dimension problem of state space, DNN is designed to classify states where the optimization policy of novel Q-Learning is set to label samples. At the same time, compared with the general RL algorithm, there is only one set of adjustable parameters, and the TQL learning proposed in this paper can further adjust the parameters according to the system requirements to further optimize the system. Thus far, the dimensions of action and state have been solved. The algorithm proposed in this paper is more flexible. Finally, the simulation result proves that the algorithm proposed in this paper is effective and feasible, and improves the convergence speed by 60% compared with the tabular QL.

## Figures and Tables

**Table 1:** Simulation Parameters.

Parameter	Value
Macro base station radius	600 m
Small base station radius	100 m
Minimum distance between small base station	40 m
Minimum distance from macro base station to small base station	70 m
System bandwidth B	10 MHz
Transmit power of MBS	Normal: 46 dBm; Sleeping: 12 dBm; $N_{TRX}=6$
Transmit power of SBS	Normal: 30 dBm; $N_{TRX}=2$
Noise power density $N_{0}$	−174 dBm
Path loss from MBS to UE	128.1 + 37.6 × $\log_{10}$(*R*[km]), *R* is the distance between MBS and UE
Path loss from SBS to UE	149.1 + 37.6 × $\log_{10}$(*R*[km]), *R* is the distance between MBS and UE
Carrier frequency	2.0 Ghz
File size	0.5 mbytes
